# Randomized controlled phase 2 trial of hydroxychloroquine in childhood interstitial lung disease

**DOI:** 10.1186/s13023-022-02399-2

**Published:** 2022-07-23

**Authors:** Matthias Griese, Matthias Kappler, Florian Stehling, Johannes Schulze, Winfried Baden, Cordula Koerner-Rettberg, Julia Carlens, Freerk Prenzel, Lutz Nährlich, Andreas Thalmeier, Daniela Sebah, Kai Kronfeld, Hans Rock, Christian Ruckes, the HCQ-study group, Margarete Olivier, Stefan Zielen, Azadeh Bagheri-Potthof, Ulrich Thome, Julia Gebhardt, Anna Mehl, Susanne Gabriele Lau, Utz Philipp, Matthias Kopp, Guido Stichtenoth, Olaf Sommerburg, Mirjam Stahl, Richard Kitz, Christoph Rietschel, Philippe Stock, Frank Ahrens, Helge Hebestreit, Florian Segerer, Folke Brinkmann, Schlegtendal Anne, Claudia Eismann, Dörthe Neuner, Sabine Witt, Meike Hengst, Maria Feilcke, Jürgen Babl, Gabriele Stauffer, Tanja Nickolay, Stanislav Gorbulev, Gisela Anthony, Linda Stöhr, Laura Vieweg, Anke Strenge-Hesse, Martin Wetzke, Elias Seidl, Nicolaus Schwerk

**Affiliations:** 1 Dr. von Hauner Children´s Hospital, University of Munich, German Center for Lung Research (DZL), Lindwurmstraße 4, 80337 Munich, Germany; 2 Uniklinikum Essen Pädiatrische Pneumologie, Kinderheilkunde III, Hufelandstr. 55, 45122 Essen, Germany; 3 Universitätsklinikum Frankfurt Klinik für Kinder- und Jugendmedizin, Pneumologie, Allergologie and Mukoviszidose, Theodor-Stern-Kai 7, 60590 Frankfurt, Germany; 4 Universitätsklinik für Kinder- und Jugendmedizin Tübingen, Hoppe-Seyler-Str. 1, 72076 Tübingen, Germany; 5 Universitätsklinik für Kinder- und Jugendmedizin im St. Josef-Hospital Bochum, Alexandrinenstraße 5, 44791 Bochum, Germany; 6 Department of Paediatric Pneumonology, Allergology and Neonatology, Hannover Medical School, German Center for Lung Research (DZL), Hannover, Germany; 7 Klinik und Poliklinik für Kinder- und Jugendmedizin der Universität Leipzig, Liebigstraße 20a, Haus 6, 04103 Leipzig, Germany; 8 Department of Pediatrics, Justus-Liebig-University Giessen, German Center for Lung Research, Universities of Giessen and Marburg Lung Center (UGMLC), Giessen, Germany; 9 Pharmacy, University Hospital of Munich, Munich, Germany; 10 IZKS, Interdisciplinary Center for Clinical Trials, University Medical Center Mainz, Mainz, Germany; 11 Central Information Office GmbH, Fronhausen, Bellnhausen, Germany

**Keywords:** chILD, Interstitial lung diseases, Hydroxychloroquine, Randomized-controlled trial

## Abstract

**Background:**

No results of controlled trials are available for any of the few treatments offered to children with interstitial lung diseases (chILD). We evaluated hydroxychloroquine (HCQ) in a phase 2, prospective, multicentre, 1:1-randomized, double-blind, placebo-controlled, parallel-group/crossover trial. HCQ (START arm) or placebo were given for 4 weeks. Then all subjects received HCQ for another 4 weeks. In the STOP arm subjects already taking HCQ were randomized to 12 weeks of HCQ or placebo (= withdrawal of HCQ). Then all subjects stopped treatment and were observed for another 12 weeks.

**Results:**

26 subjects were included in the START arm, 9 in the STOP arm, of these four subjects participated in both arms. The primary endpoint, presence or absence of a response to treatment, assessed as oxygenation (calculated from a change in transcutaneous O_2_-saturation of ≥ 5%, respiratory rate ≥ 20% or level of respiratory support), did not differ between placebo and HCQ groups. Secondary endpoints including change of O_2_-saturation ≥ 3%, health related quality of life, pulmonary function and 6-min-walk-test distance, were not different between groups. Finally combining all placebo and all HCQ treatment periods did not identify significant treatment effects. Overall effect sizes were small. HCQ was well tolerated, adverse events were not different between placebo and HCQ.

**Conclusions:**

Acknowledging important shortcomings of the study, including a small study population, the treatment duration, lack of outcomes like lung function testing below age of 6 years, the small effect size of HCQ treatment observed requires careful reassessments of prescriptions in everyday practice (EudraCT-Nr.: 2013-003714-40, www.clinicaltrialsregister.eu, registered 02.07.2013).

*Registration* The study was registered on 2 July 2013 (Eudra-CT Number: 2013-003714-40), whereas the approval by BfArM was received 24.11.2014, followed by the approval by the lead EC of the University Hospital Munich on 20.01.2015. At clinicaltrials.gov the trial was additionally registered on November 8, 2015 (NCT02615938).

## Background

Interstitial lung diseases in children (chILD) are a large and heterogeneous group of rare and chronic conditions, often related to inflammatory and sometimes to fibrosing disease processes [1]. They mainly affect the lung parenchyma, cause severe morbidity in a relevant proportion of affected individuals, and have a mortality of about 15% [2].

Up to date no proven anti-inflammatory or anti-fibrotic treatments of these conditions are available, as no prospective trials on efficacy and safety of treatments in chILD have ever been performed [3]. The few pharmacological options used are based on anecdotal experience and small case collections. For several decades, besides systemic glucocorticosteroids, most commonly hydroxychloroquine (HCQ) or chloroquine have been applied [4].

HCQ can inhibit the production of inflammatory cytokines (e.g. IL-1, IL-6, TNFα and INFγ) and the degradation of intracellular cargo via the autophagy pathway [5]. It can interfere with aberrantly produced proteins in cells affected by pathogenic variants in the genes for surfactant protein C [6], ABCA3 [7], COPA [8] and others. These proteins are degraded via the lysosomal pathway or may be presented as autoantigens and drive undesirable inflammatory and pro-fibrotic immune responses [5]. This interference might explain the favourable clinical responses to HCQ or chloroquine reported in cases and small series of children with interstitial lung disease [4]. As these drugs are often given for many years and potentially cause severe side effects, there is an urgent need for evidence [9, 10]. Therefore, the European-wide project chILD-EU initiated a randomized phase 2a study of HCQ in chILD, evaluating the efficacy and safety of the mid-term use of HCQ [2].

In this study we focused on the efficacy and safety of hydroxychloroquine (HCQ) in patients with chILD and a lung histology pattern of chronic pneumonitis of infancy, non-specific interstitial pneumonitis (NSIP), desquamative interstitial pneumonitis, microscopic alveolar proteinosis or cholesterol pneumonitis, pulmonary hemosiderosis, follicular bronchiolitis and lymphocytic interstitial pneumonitis, as well as on chILD caused by mutations in SFTPC, ABCA3, NKX2.1, TBX4, or COPA.

## Methods

### Trial design and participants

**Fig. 1:**
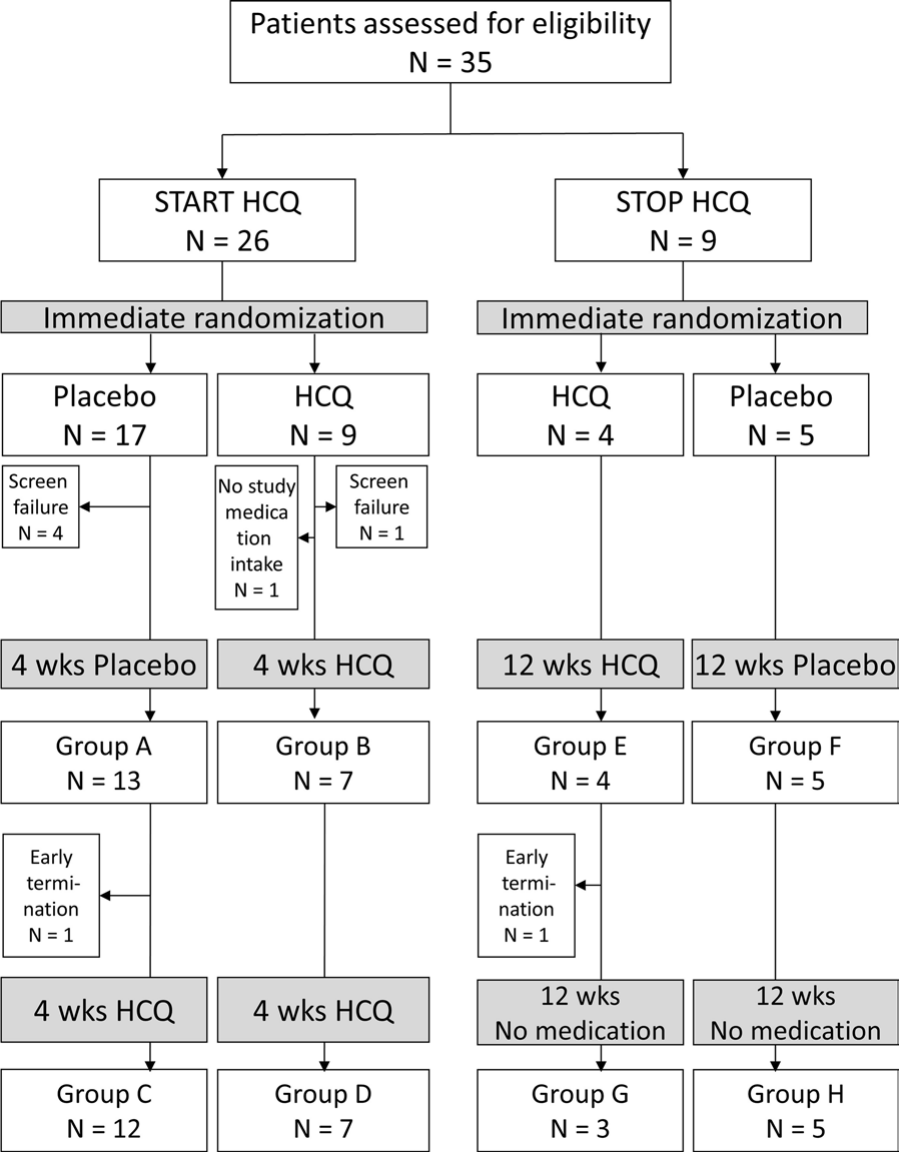
Flow Diagram (CONSORT) and trial design

This study was a prospective, multicentre, 1:1 randomized, double-blind, placebo-controlled parallel-group/crossover phase 2 clinical trial. The study design was previously described in detail [11]. In summary, subjects with a chronic (≥ 3 weeks’ duration) diffuse parenchymal lung disease and eligible for treatment with HCQ (START arm) or withdrawal of HCQ (STOP arm) were asked to participate. We assessed in- and exclusion criteria (Tabs. 2 and 3 in [11]), obtained written consent and for logistic reasons, i.e. personalized preparation of weight adapted study medication, randomized each subject after screening evaluation (Fig. 1). In the START arm subjects were allocated to 4 weeks of placebo (group A) or HCQ (group B; receiving 10 mg/kg bodyweight/d during the first week, then 6.5 mg/kg/d orally in the evening). Then subjects from group A were switched to HCQ for 4 weeks (groups C), while group B remained on HCQ for another 4 weeks (group D). In the STOP arm subjects already taking HCQ for at least 3 months were randomized into parallel groups treated with HCQ (group E) at the dose they were already on or with placebo (i.e. means withdrawal of HCQ, group F). After 12 weeks all subjects stopped medication and moved into open observation for another 12 weeks (groups G and H, Fig. 1). Each subject could participate in each arm only once; arms were initiated in any sequence (see Fig. 1 for study scheme).

Subjects had to be clinically stable between screening and baseline visit. All subjects were included in the registry and the diagnosis was verified by a structured peer review process (2). We included seven children with ABCA3 deficiency, six with surfactant protein C deficiency, two with NKX2.1 deficiency, three with COPA syndrome and one with TBX4 deficiency and fibrosing filamin A deficiency, respectively. Four subjects without genetic proof of a lung disease had a NSIP histologic pattern, one subject each had the histological pattern of pulmonary alveolar proteinosis, pleuroparenchymal fibroelastosis, and idiopathic desquamative interstitial pneumonitis. One case each of chronic tachypnea of infancy, nodular lymphoid hyperplasia of the lung, fibrosing hyper IgG4-syndrome, and sarcoidosis were also included. Two subjects were diagnosed as idiopathic pulmonary hemosiderosis (IPH) and two others suffered from chronic diffuse parenchymal lung disease, which could not further characterized. Active study centers including patients were the University Children´s Hospitals at Munich, Hannover, Essen, Frankfurt, Tübingen and Bochum.

### Outcomes

The primary study endpoint was the presence or absence of a response to treatment. A responder was defined as a subject who had a predefined change in oxygenation at rest and calm wakefulness. Oxygenation was assessed by measurement of the transcutaneous O_2_-saturation by pulse oximetry, the respiratory support level necessary to achieve this level and respiratory rate. Different level of respiratory support were defined as invasive ventilation, non-invasive ventilation, high-flow O_2_ nasal cannula, low flow O_2_ by prongs/mask and room air.

In subjects who were on low flow oxygen O_2_-saturation was measured after O_2_ withdrawal for at least 5 min. In patients included in the START group and who were off oxygen or on low flow oxygen at study entry, response was defined as an increase of oxygen saturation by ≥ 5% and/or a decrease of respiratory rate at rest ≥ 20% compared to baseline, assessed under room air conditions. In patients with a higher level of respiratory support at the time of inclusion, response was defined as a sustained decrease of the respiratory support compared to baseline. For STOP patients O_2_-saturation had to decrease by ≥ 5% or the respiratory rate to increase by ≥ 20%, assessed under room air conditions, or the subject needed an increased level of respiratory support.

Secondary endpoints were exploratory and included among others the modified definition of a responder, as a subject who had a change of the oxygenation by 3%, changes in O_2_-saturation in room air, respiratory rate, health related quality of life (HrQoL) [12], BMI percentile, pulmonary function [13] and 6-min walk test (6MWT) distance.

Safety monitoring included adverse events (AEs), clinical laboratory values (differential blood count, glutamic oxaloacetic transaminase (GOT), glutamate-pyruvate transaminase (GPT), gamma glutamyl transpeptidase (gGT), creatinine, lactate dehydrogenase (LDH), potassium, creatine kinase, blood glucose levels), HCQ steady-state drug level [14], electrocardiography, echocardiography and repeated ophthalmological examinations.

### Statistical methods

As the study was exploratory there was no formal sample size calculation. All subjects randomized were included in the intention to treat analysis (ITT), which was defined as the primary analysis population. Statistical sensitivity analysis were planned for the combined analysis of all patients in the START and STOP arms. However, depending on actual recruitment structure, the assumption of independence of subjects participating in both study parts might not be fully justified. Those subjects receiving at least one dose of study drug defined the safety population. Data are given as mean and standard deviation or frequency of events. Changes with treatment were calculated and compared between placebo and HCQ groups. The groups are defined in Fig. 1. Continuous variables were compared by unpaired or paired t-tests, responder frequencies by Fisher exact tests or Mc Nemar test. Bonferoni corrections were made for using a variable repetitively; a level of *P* < 0.05 was considered significant. To estimate the magnitude of the treatment effects for independent responders odds ratios with 95%-confidence intervals, for dependent responders Kappa coefficient with 95%-confidence intervals and for continuous variables effect sizes defined as the changes to baseline of both treatments divided by the pooled standard deviation of the changes to baseline with 95%-confidence intervals were calculated from treatment effects under HCQ or placebo.

## Results

### Enrollment and baseline characteristics of the subjects

35 subjects were assessed for eligibility, 26 for the HCQ START arm and 9 for the HCQ STOP arm (Fig. 1). There were five screening failures and one drop out before drug intake. On study medication, another two subjects ended the trial prematurely, one in the START and one in the STOP arm. Only four subjects were included in both arms; we considered these subjects as independent individuals. The trial was terminated after 3.8 years of recruitment after a temporary interruption due to a competent authority inspection, associated with losses of time and resources of more than one and a half years, resulting in insufficient capacity thereafter to continue. The baseline data of the subjects included were not different between the groups and characteristic for children affected by interstitial lung disease (Table 1).

**Table 1:** Baseline data

	Start		Stop	
	HCQ	Placebo	HCQ	Placebo
Subjects included [n]/male	9/2	17/7	4/2	5/2
Age (y)	7.8 (5.6)	9.0 (6.8)	9.3 (6.3)	8.2 (9.0)
O2-sat, in room air (%)	93.9 (5.8)	92.6 (6.7)	93.7 (5.1)	94.8 (4.0)
Resp. rate in room air (/min)	31.0 (13.6)	33.7 (15.7)	23.3 (3.1)	34.4 (17.3)
QoL chILD specific score	66.9 (24.5)	71.2 (17.9)	75.9 (19.3)	54.9 (31.8)
QoL total score	58.3 (24.7)	65.2 (17.2)	63.3 (14.9)	72.1 (18.7)
BMI percentile	29.2 (37.5)	34.1 (34.4)	20.8 (25.4)	18.7 (15.9)
LDH (U/l)	322.5 (91.2)	351.1 (91.0)	330.5 (136.3)	292 (15.0)
FVC (% predicted)	44.3 (21.2)	46.7 (20.8)	51.7 (4.0)	76.7 (22.5)
FEV1 (% predicted)	43.8 (19.7)	44.6 (19.2)	50.7 (3.2)	71.0 (22.5)
MMEF25-75 (% predicted)	76.7 (31.3)	71.4 (39.0)	78.0 (31.2)	81.7 (30.2)
6MWT distance (% predicted)	74.2 (28.4)	58.3 (23.1)	58.3 (15.0)	88.0 (19.9)
Stable co-medication (%)				
Prednisolon	3 (33)	8 (47)	0 (0)	1 (20)
Methotrexate/Azathioprine	1 (11)	5 (29)	0 (0)	1 (20)
Azithromycine	2 (22)	4 (23)	2 (50)	1 (20)
Sildenafil/Bosentan	0 (0)	7 (41)	2 (50)	2 (40)
HCQ whole blood level at baseline (ng/ml)	0 (0)	0 (0)	432 (709)	345 (370)

Data are given as mean (SD) or number of subjects (%). No differences were detected between the groups

*nd* not done, *6MWT* 6 Minute walk test

### Outcome- efficacy results

The primary endpoint, the presence or absence of a response to the treatment, did not differ between placebo and HCQ groups (Table 2). In the START arm there were no responders to placebo treatment (group A), as were for HCQ in the parallel group (group B). After switching from placebo to HCQ three responder were noted (group C). Combining the two HCQ treatment groups B and C did not change the result. We obtained similar results in the STOP arm: no responder to placebo treatment (= withdrawal of HCQ) (group F), as for HCQ treatment (= continuation of HCQ) in the parallel group (group E). After open label observation (= no medication, no med.; = withdrawal of HCQ) (group G) one responder was noted (Table 2). To increase the sensitivity we explored an adapted responder definition. Based on the minimal important difference for O_2_-saturation we used a 3% threshold for change in oxygenation. Again, we observed no differences (Table 2). To describe the size of the treatment effects obtained we calculated the odds ratios of the responders under placebo and under HCQ; these were around one, negative or could not be calculated, as there were zero responder.

**Table 2:** Number of responders to treatment

Variable	Start					Stop				
	Group (treatment)	Yes (n)	No (n)	Comparison to A (placebo 4wks); *P*	Odds ratio/Kappa coefficient (95% CI)	Group (treatment)	Yes (n)	No (n)	Comparison to E (HCQ 4wks); *P*	Odds ratio/ Kappa coefficient (95% CI)
Responders to treatment (defined per protocol)	A (placebo 4wks)	0	13			E (HCQ 12wks)	0	3		
	B (HCQ 4wks)	0	7	1.000^a^	Not appl^c^	F (placebo 12wks)	0	5	1.000^a^	Not appl^c^
	C (HCQ 4wks)	3	9	Not appl^b,c^	Not appl^c^	G (no med. 12wks)	1	2	Not appl^b,c^	Not appl^c^
	B + C (combined)	3	16	0.253^a^	Not appl^c^	F + G combined	1	7	1.000^a^	Not appl^c^
Responders to treatment (defined by MID)	A (placebo 4wks)	2	11			E (HCQ 12wks)	0	3		
	B (HCQ 4wks)	1	5	1.000^a^	1.100 (0.079–15.16)	F (placebo 12wks)	3	2	0.196^a^	Not appl^c^
	C (HCQ 4wks)	2	10	1.000^b^	− 0.2 (− 0.340–0.008)	G (no med. 12wks)	1	2	Not appl^b,c^	Not appl^c^
	B + C (combined)	3	15	1.000^a^	1.100 (0.156–7.740)	F + G (combined)	4	4	0.236^a^	Not appl^c^

Data are given as number (n) of patients responding or not. MID = minimum important difference (defined as 3% change in O_2_-saturation)

Remark: in group B “Responders to treatment (defined by MID)” has one subject less than “Responders to treatment (defined per protocol)”, because in that case the O2 saturation in room air was missing. no med. = no medication, means withdrawal of HCQ

^a^Fisher exact test

^b^McNemar test for paired samples and compares A and C or E and G

^c^not appl = cannot be calculated if in any group no responder exists (i.e. no odds exists for independent groups or no disconcordant pairs could be determined for dependent groups). *P* – values are given and a *P* < 0.05 was considered significant

Using the per protocol definition, the 4 patients who responded to HCQ or HCQ withdrawal had the diagnosis: 2 × ABCA3 deficiency, 1 × SP-C deficiency and 1 × Filamin A deficiency; no patients responded to placebo. Using the MID definition, the 7 patients who responded to HCQ (groups B, C) or HCQ withdrawal (groups F, G) had the following diagnoses: 4 × ABCA3 deficiency, 1 × NSIP, 1 × SP-C deficiency and 1 × Filamin A deficiency. The 2 responders to placebo (group A) had the following diagnoses: 1 × COPA-syndrome, 1 × cNSIP

For all the continuous variables, we calculated the changes for the different treatment groups (Table 3). Absolute changes in O_2_-saturation, respiratory rate, HrQoL and in pulmonary function or exercise tests, were not significantly different with treatment, neither in the parallel group (A vs. B), the paired (A vs. C) nor the combined (A vs. B + C) comparisons for the START arms or the STOP arms. Of interest in the START arm, BMI percentile dropped with HCQ treatment (borderline level of significance (Table 3)). Thus we did not observe significant differences with interventions.

**Table 3:** Absolute changes from baseline of secondary outcomes

Variable	Start				Stop			
	Group (treatment)	Absolute changes from baseline	Comparison to A (placebo 4wks); *P*	Effect size (95% CI)	Group (treatment)	Absolute changes from baseline	Comparison to E (HCQ 4wks); *P*	Effect size (95% CI)
O2-sat in room air (%)	A (placebo 4wks)	− 0.1 (2.4)			E (HCQ 12wks)	0.3 (1.2)		
	B (HCQ 4wks)	− 0.6 (3.3)	0.757	− 0.2 (− 1.2−0.8)	F (placebo 12wks)	− 2.4 (2.7)	0.097	− 1.2 (− 2.7–0.4)
	C (HCQ 4wks)	1.8 (3.5)	0.229	0.6 (− 0.2−1.4)	G (no med. 12wks)	− 3.7 (6.4)	0.427	− 0.9 (− 2.5−0.8)
	B + C (combined)	1.1 (3.5)	0.300	0.4 (− 0.3﻿−1.1)	F + G combined	− 2.9 (4)	0.071	− 0.9 (− 2.3−0.5)
Resp. rate in room air ( /min)	A (placebo 4wks)	1.2 (5.4)			E (HCQ 12wks)	− 2.7 (1.2)		
	B (HCQ 4wks)	0.2 (0.4)	0.543	− 0.2 (− 1.2−0.8)	F (placebo 12wks)	− 5.4 (9.4)	0.553	− 0.4 (− 1.8−1.1)
	C (HCQ 4wks)	− 1.3 (5.6)	0.421	− 0.5 (− 1.2−0.3)	G (no med. 12wks)	7.3 (5)	0.102	2.8 (0.5−5)
	B + C (combined)	− 0.9 (4.8)	0.289	− 0.4 (− 1.1−0.3)	F + G combined	− 0.6 (10)	0.589	0.2 (− 1.1−1.6)
Quality of life (chILD specific)	A (placebo 4wks)	6.9 (9.6)			E (HCQ 12wks)	− 5.7 (11.2)		
	B (HCQ 4wks)	0.1 (3.18)	0.178	− 0.8 (− 2.4−0.9)	F (placebo 12wks)	7.5 (2.3)	0.338	1.6 (− 0.6−3.9)
	C (HCQ 4wks)	− 3.2 (4.8)	0.110	− 1.3 (− 2.6–− 0.1)	G (no med. 12wks)	2.3 (3.2)	0.398	1.0 (− 1.1−3.0)
	B + C (combined)	− 2.4 (4.5)	0.064	− 1.3 (− 2.5–−0.2)	F + G combined	4.9 (3.8)	0.403	1.6 (− 0.3−﻿3.6)
Quality of life (total score)	A (placebo 4wks)	0.4 (6.9)			E (HCQ 12wks)	9.2 (5.4)		
	B (HCQ 4wks)	0.3 (1.2)	0.965	0 (− 1.6–1.6)	F (placebo 12wks)	− 9.5 (8.8)	0.149	− 2.6 (− 5.2−0.1)
	C (HCQ 4wks)	4.8 (4.3)	0.374	0.8 (− 0.4–1.9)	G (no med. 12wks)	− 1.7 (2.3)	0.296	− 2.6 (− 5.3–0.0)
	B + C (combined)	3.7 (4.3)	0.333	0.6 (− 0.5–1.7)	F + G combined	− 5.6 (6.9)	0.072	− 2.3 (− 4.4– -0.1)
BMI percentile	A (placebo 4wks)	7.5 (11.7)			E (HCQ 12wks)	− 3.3 (7.9)		
	B (HCQ 4wks)	− 1.8 (5.1)	0.024	− 0.9 (− 1.9-0)	F (placebo 12wks)	2.2 (11.4)	0.458	0.5 (− 0.9−2.0)
	C (HCQ 4wks)	− 1.6 (8.1)	0.045	− 0.9 (− 1.7–−0.1)	G (no med. 12wks)	13.3 (20.9)	0.422	1.1 (− 0.7–2.8)
	B + C (combined)	− 1.7 (7.1)	0.020	− 1.0 (− 1.7−–0.3)	F + G combined	6.4 (15.2)	0.213	0.7 (− 0.7−2.1)
LDH (U/ml)	A (placebo 4wks)	− 28.2 (53.7)			E (HCQ 12wks)	34.5 (0.7)		
	B (HCQ 4wks)	43.0 (61)	0.063	1.3 (0.1−2.5)	F (placebo 12wks)	9.3 (65.5)	0.574	− 0.5 (− 2.3−1.3)
	C (HCQ 4wks)	11.3 (67)	0.410	0.6 (− 0.3−﻿1.6)	G (no med. 12wks)	Not done	n.appl	n.appl
	B + C (combined)	21.9 (64.7)	0.055	0.8 (0−1.7)	F + G combined	9.3 (65.5)	0.574	− 0.5 (− 2.3−1.3)
FVC (% pred)	A (placebo 4wks)	0.0 (3.7)			E (HCQ 12wks)	2.3 (7.6)		
	B (HCQ 4wks)	2.5 (13)	0.730	0.3 (− 0.9−1.5)	F (placebo 12wks)	0.5 (2.1)	0.724	− 0.3 (− 2.1−1.5)
	C (HCQ 4wks)	10.6 (19.7)	0.170	0.7 (− 0.3−1.8)	G (no med. 12wks)	− 1.3 (5.1)	0.487	− 0.6 (− 2.2−1.1)
	B + C (combined)	7.9 (17.6)	0.157	0.6 (− 0.3−1.5)	F + G combined	− 0.6 (3.9)	0.583	− 0.5 (− 2.0−0.9)
FEV1 (% pred)	A (placebo 4wks)	0.6 (4.1)			E (HCQ 12wks)	0.0 (4.4)		
	B (HCQ 4wks)	2.0 (13.1)	0.850	0.2 (− 1−1.4)	F (placebo 12wks)	− 0.5 (0.7)	0.862	− 0.1 (− 1.9–1.7)
	C (HCQ 4wks)	9.0 (18.6)	0.258	0.6 (− 0.4−1.6)	G (no med. 12wks)	− 1.0 (4.4)	0.840	− 0.2 (− 1.8−1.4)
	B + C (combined)	6.7 (16.7)	0.251	0.5 (− 0.4−1.4)	F + G combined	− 0.8 (3.1)	0.798	− 0.2 (− 1.7−1.2)
MMEF25-75 (% pred)	A (placebo 4wks)	3.0 (13.1)			E (HCQ 12wks)	− 8.7 (14.2)		
	B (HCQ 4wks)	− 14.8 (15.2)	0.010	− 1.3 (− 2.6−0)	F (placebo 12wks)	− 7.0 (1.4)	0.858	0.1 (− 1.6−1.9)
	C (HCQ 4wks)	− 2.6 (8.6)	0.533	− 0.5 (− 1.5−0.5)	G (no med. 12wks)	− 11.3 (7.8)	0.661	− 0.2 (− 1.8−1.4)
	B + C (combined)	− 7.0 (12.3)	0.114	− 0.8 (− 1.7−0.2)	F + G combined	− 9.6 (6)	0.922	− 0.1 (− 1.5−1.3)
6MWT distance (% pred)	A (placebo 4wks)	− 0.8 (3.6)			E (HCQ 12wks)	0.3 (8.5)		
	B (HCQ 4wks)	− 31.0 (n.appl.)	n.appl	− 8.4 (− 14– −2.7)	F (placebo 12wks)	− 0.5 (2.1)	0.884	− 0.1 (− 1.9−1.7)
	C (HCQ 4wks)	0.8 (2.1)	0.576	0.5 (− 0.9−2)	G (no med. 12wks)	8.7 (12.6)	0.563	0.8 (− 0.9−2.4)
	B + C (combined)	− 5.6 (14.3)	0.501	− 0.4 (− 1.8−0.9)	F + G combined	5.0 (10.3)	0.518	0.5 (− 1.0−1.9)

Data are given as mean (SD); the number of subjects in each group is indicated in Fig. 1. In START or STOP for each parameter 3 comparisons were made and thus after Bonferroni correction a *P* < 0.017 considered significant

*6 MWT* 6 min walk test, *QoL* Quality of life questionnaire, *no med*. no medication, means withdrawal of HCQ (comparable to placebo), *n.a*. not available, *n.appl*. not applicable

### Sensitivity analysis

In an exploratory sensitivity analysis we combined all treatment periods with placebo and all periods with HCQ from START with those from STOP, the latter adjusted by multiplying for withdrawal by -1 (Table 4) and using data from START and STOP independently. Again, we did not identify consistent treatment effects of HCQ for the primary and the secondary endpoints (Responder (MID), O_2_-saturation, respiratory rate in room air, and FVC absolute change). Significant decreases of HrQoL, assessed as total score and BMI percentile in the HCQ treated groups were noted; effect sizes were again small (Table 4).

**Table 4:** Changes observed from START and those from STOP treatment groups were combined to explore maximum number of treatment effects

	“HCQ-effect”	“Placebo-effect”	*P*	Odds ratio/Kappa coefficient /Effect size (95% CI)
Groups combined	B, F, C, G	A, E		
Responder (protocol definition) [yes, no]	4, 23	0, 16	0.106	Not appl^a,b^
Responder (minimum important difference) [yes, no]	7, 19	2, 14	0.269	2.6 (0.5–14.4)^b^
O2 saturation (%) in room air	1.6 (3.7) 25	− 0.1 (2.2) 16	0.062	0.5 (− 0.1–1.2)
Respiratory rate (/min) in room air	− 0.4 (6.7) 26	1.4 (4.9) 16	0.306	− 0.3 (− 0.9 − 0.3)
QoL chILD specific	− 3.2 (4.3) 12	6.6 (9.1) 8	0.019	− 1.5 (− 2.5–− 0.5)
QoL total score	4.3 (5.1) 12	− 2.0 (7.6) 8	0.063	1.0 (0.1–2.0)
BMI percentile	− 3.0 (10.0) 28	6.7 (10.9) 16	0.007	− 0.9 (− 1.6–− 0.3)
FVC absolute change (% predicted)	5.8 (15.1) 17	− 0.6 (4.7) 11	0.119	0.5 (− 0.2–1.3)

Data are number of subjects for responder/non-responder and means (SD) n for the other variables. Changes in Stop were multiplied by -1 before combining. After Bonferroni correction for multiple (four) comparisons, a *P* value < 0.0125 was considered significant

^a^not appl = cannot be calculated if in any group no responder exists (i.e. no odds exists for independent groups)

^b^Odds ratios and Kappa coefficients were calculated for responder evaluation, see also Table 2

### Outcome: safety

Adherence to the study medication was 91% in both, START and STOP arms, and were not different between placebo and HCQ treatment. In general, the study drug was well tolerated. In almost all subjects, adverse events were observed (Table 5). These were primarily gastrointestinal or respiratory infections. There were no differences in frequency of AEs between placebo or HCQ groups. During the entire study, we observed only one serious adverse event. This occurred in the placebo group in a sick infant on non-invasive respiratory support who had to be intubated due to an intercurrent respiratory infection. The event resolved completely. Overall, the AE were characterized by the morbidity of the study population and the known side effect spectrum of the study drug. HCQ whole blood levels, measured at the end of the study in the START subjects did not differ from baseline levels in patients who were to discontinue HCQ (mean dose 6 mg/kg body-weight) (Table 1). This suggested that a steady state was achieved in blood. Of interest, intra-individual values were rather constant, whereas, inter-individual levels varied considerably.

**Table 5:** Adverse events during the study in the safety population. Given are numbers of subjects with events and number of events (absolute/% of total)

	Start				Stop			
	HCQ		Placebo		HCQ		Placebo	
	Subjects	Events	Subjects	Events	Subjects	Events	Subjects	Events
Subjects eligible for AES (after drugging)	7		13		4		5	
Subjects with AEs or Numbers of events	7	11	12	29	4	10	5	17
Any AE	6 (75%)	11 (100%)	10 (83%)	29 (100%)	3 (75%)	10 (100%)	5 (100%)	17 (100%)
Any related AEs	1 (13%)	3 (27%)	1 (8%)	2 (7%)	–	–	–	–
Any serious AEs/deaths	–	–	1 (8%)	1 (3%)	–	–	–	–
Any AEs leading to premature study discontinuation	–	–	1 (8%)	1 (3%)	–	–	–	–
Details on AEs*								
Infections (sinusitis, otitis, upper/lower respiratory tract infection)	3 (43%)	4 (36%)	9 (75%)	15 (52%)	2 (50%)	6 (60%)	4 (80%)	13 (76%)
Blood (White blood cell disorder)	–	–	1 (8%)	1 (4%)	–	–	–	–
Psychiatric disorders (restlessness)	–	–	1 (8%)	1 (4%)	–	–	–	–
Nervous system disorders	–	–	1 (8%)	1 (4%)	–	–	–	–
Eye disorders (Cataract)	–	–	1 (8%)	1 (4%)	–	–	–	–
Respiratory disorders (cough, pain)	1 (14%)	2 (18%)	2 (17%)	3 (10%)	2 (50%)	2 (20%)	1 (20%)	1 (6%)
Gastrointestinal disorders (constipation, stomatitis)	2 (29%)	4 (36%)	2 (17%)	3 (10%)	2 (50%)	2 (20%)	1 (20%)	1 (6%)
Musculoskeletal and connective tissue disorders (Arthralgia)	–	–	1 (8%)	2 (7%)	–	–	1 (20%)	1 (6%)
General disorders (exercise tolerance decreased, hyperthermia malignant, pyrexia)	1 (14%)	1 (9%)	1 (8%)	1 (4%)	–	–	1 (20%)	1 (6%)

*Same subject can have several different events

## Discussion

In this double blind, randomized controlled exploratory phase 2 trial in paediatric patients with chILD, we evaluated the efficacy and safety of the use of HCQ. The primary outcome was change in oxygenation, determined from O_2_-saturation at room air, respiratory rate or a change in respiratory support. The results of these and the other key secondary endpoints did not differ between HCQ and placebo treatment periods. Adherence to the treatment was good, the drug was well-tolerated and appeared save.

The authors were aware that this investigator-initiated study in a group of ultra-rare conditions might have difficulties recruiting subjects, even in centers specialized to treat such conditions. Therefore, we classified the study as exploratory and developed a design, which allowed including many potential participants by close alignment of study procedures to everyday patient management. To treat all participants with active drug, we implemented a switch from placebo to HCQ for all subjects. Similarly, a controlled withdrawal of HCQ was ensured in all. In an exploratory statistical sensitivity analysis, we combined all observed effects in treatment and placebo periods to maximize contrasts. Although the drug had been widely used in children, the execution of the study was monitored very closely. With all these measures we took as many precautions as possible to optimize the study design, the execution of the research and the validity of the study results.

After a routine inspection by the authorities and the identification of recoverable findings, the study was temporarily suspended. Whereas we duly addressed all issues raised on the patient and center level, including shortcomings in storage of study medication and documents, documentation logs and consenting procedures, structural improvements beyond the sponsor delegated person´s liability would take longer. These involved the University hospital´s overall study structure and included defects in sponsor oversight from non-uniform SOPs, structural control deficits, non-systematic electronic case-report form user right management, missing risk analysis plan, and insufficient change control management. The study was already recruiting for almost 4 years and 35 subjects were included. In particular as the COVID-19 pandemic spread, we decided closing the trial due to insufficient capacities to continue. All data were extensively and carefully reviewed by monitors on site and remotely, where feasible. Additionally, we assessed data completeness and internal consistency by central monitoring before data base closure. Based on these suppositions, we classified the quality of the trial and obtained data as well suited for analysis.

The dichotomous primary endpoint is appropriately expressed as odds ratio of the responders under placebo and under HCQ. In both the, START and STOP arms, there were no responders in the HCQ and placebo groups. Thus, no ratio could be calculated. In an exploratory analysis we reduced the threshold for response by using a 3% change in O_2_-saturation. Now in the START arm odds ratios around 1 and a small kappa coefficient could be calculated, not supporting a treatment response to HCQ. To increase the study power as much as possible, we combined all treatment groups, i.e. all 27 “HCQ treatments” and the 16 “placebo treatments” (Table 4). Nevertheless, odds ratios and kappa coefficients of responders defined by protocol or MID definition, as well as the effect sizes of the relevant secondary outcomes lung function and quality of life were all marginal, most often spanning zero and clinically negligible.

When reflecting about the response rates we hypothesized that about 70% of the subjects would respond to HCQ and 35% to placebo [15]. These assumptions were based on our comprehensive literature review which identified 85 patients treated with HCQ between 1984 and 2013 who were found to have a 41% response rate [4]. However, it must be considered that in those publications “response” was primarily a clinical impression and not defined precisely. Complicating, other medications like systemic steroids were often started at the same time as HCQ. Only 16 patients were treated exclusively with HCQ and of these, 88% (14 patients) responded [4]. Such a high response rate might be due in part to a publication bias for positive studies and is very likely further skewed by uncontrolled treatment conditions, undefined response criteria and retrospective analyses. However, if such a high response rate was real, it was very unlikely to have been missed in this study, as only 32 patients would be needed to detect the treatment difference in a post-study calculation using a power of 80% and an alpha levels of 5%. Having all this in mind, we must be aware that there is a chance to incorrectly accept the null hypothesis and falsely rate this treatment negative.

Additionally to the limitations listed further issues need to be considered. First, based on a Delphi process involving chILD experts [3], we chose a treatment duration of four weeks to determine the response to the study drug. However also after 8 weeks of HCQ treatment (Group D) there were no more responders (data not shown). Longer term treatments could be investigated in future trials. Second, the wide range of chILD diagnoses reported to respond to HCQ [4] and included into the study could mask strong responses in certain conditions. However we did not identify response clusters in molecularly or histologically defined chILD sub-entities (data not shown). Whereas in adults with interstitial lung disease, lumping approaches to assess drug effects are common practice [16], a gene and mutation specific treatment of patients based on strong in vitro evidence was very successful in cystic fibrosis [17, 18]. Unfortunately, to date there is no relevant in vitro test for HCQ linking it to lung disease [5]. In ABCA3 deficiency, an important chILD subgroup, this approach has been shown to be effective for some compounds [19, 20].

Currently an industry sponsored phase 3 trial of nintedanib in children with fibrosing chILD (ClinicalTrials.gov: NCT04093024) is ongoing, aiming to include at least 30 patients [21]. This points out the extraordinary logistic effort and financial power necessary to recruit such a relatively small number of subjects in this condition.

## Conclusions

For the first time this study has generated controlled evidence on the effect size of HCQ treatment in chILD. Disappointingly and considering the many precautions indicated above, we suggest that the past optimistic appraisal of HCQ in chILD needs to be revised. In every instance it is prescribed to children its efficacy should be assessed repeatedly and quantitatively, the length of treatment needs to be limited to reasonable periods, and the patient is best followed in a chILD-register for future data aggregation [2].

## Data Availability

The study protocol was published [11] and is available upon request from the corresponding author. The authors make the primary data available to other research groups on reasonable request.

## Abbreviations

ABCA3: ATP-binding cassette transporter A3
chILD: Children’s interstitial lung disease
COPA: Coatomer protein complex, alpha subunit
HCQ: Hydroxychloroquine
NKX2.1: NK2 homeobox 1
NSIP: Non-specific interstitial pneumonitis (NSIP)
SFTPC: Surfactant, pulmonary associated protein C
SP-C: Surfactant protein C
TBX4: T-box 4

## References

[1] Nathan N, Sileo C, Thouvenin G (2019). Pulmonary fibrosis in children. J Clin Med.

[2] Griese M, Seidl E, Hengst M (2018). International management platform for children’s interstitial lung disease (chILD-EU). Thorax.

[3] Bush A, Cunningham S, De Blic J (2015). European protocols for the diagnosis and initial treatment of interstitial lung disease in children. Thorax.

[4] Braun S, Ferner M, Kronfeld K (2015). Hydroxychloroquine in children with interstitial (diffuse parenchymal) lung diseases. Pediatr Pulmonol.

[5] Schrezenmeier E, Dörner T (2020). Mechanisms of action of hydroxychloroquine and chloroquine: implications for rheumatology. Nat Rev Rheumatol.

[6] Thurm T, Kaltenborn E, Kern S (2013). SFTPC mutations cause SP-C degradation and aggregate formation without increasing ER stress. Eur J Clin Invest.

[7] Tomer Y, Wambach J, Knudsen L (2021). The common ABCA3E292V variant disrupts AT2 cell quality control and increases susceptibility to lung injury and aberrant remodeling. Am J Physiol-Lung Cell Mol Physiol.

[8] Kumrah R, Mathew B, Pandiarajan Vignesh SS (2019). Genetics of COPA syndrome. Appl Clin Genet.

[9] Williamson M, Wallis C (2014). Ten-year follow up of hydroxychloroquine treatment for ABCA3 deficiency. Pediatr Pulmonol.

[10] Avital A, Hevroni A, Godfrey S (2014). Natural history of five children with surfactant protein C mutations and interstitial lung disease. Pediatr Pulmonol.

[11] Griese M, Köhler M, Witt S (2020). Prospective evaluation of hydroxychloroquine in pediatric interstitial lung diseases: study protocol for an investigator-initiated, randomized controlled, parallel-group clinical trial. Trials.

[12] Niemitz M, Schwerk N, Goldbeck L (2018). Development and validation of a health-related quality of life questionnaire for pediatric patients with interstitial lung disease. Pediatr Pulmonol.

[13] Quanjer PH, Brazzale DJ, Boros PW (2013). Implications of adopting the Global Lungs Initiative 2012 all-age reference equations for spirometry. Eur Respir J.

[14] Zahr N, Urien S, Funck-Brentano C (2021). Evaluation of hydroxychloroquine blood concentrations and effects in childhood-onset systemic lupus erythematosus. Pharmaceuticals.

[15] Walach H, Sadaghiani C, Dehm C (2005). The therapeutic effect of clinical trials: understanding placebo response rates in clinical trials–a secondary analysis. BMC Med Res Methodol.

[16] Ryerson CJ (2019). Lumpers versus splitters: What to do with suspected idiopathic pulmonary fibrosis?. Respirology.

[17] Middleton PG, Mall MA, Dřevínek P (2019). Elexacaftor–tezacaftor–ivacaftor for cystic fibrosis with a single Phe508del allele. N Engl J Med.

[18] Griese M, Costa S, Linnemann RW (2021). Safety and efficacy of elexacaftor/tezacaftor/ivacaftor for 24 weeks or longer in people with cystic fibrosis and one or more F508del alleles: interim results of an open-label phase 3 clinical trial. Am J Respir Crit Care Med.

[19] Kinting S, Höppner S, Schindlbeck U (2018). Functional rescue of misfolding ABCA3 mutations by small molecular correctors. Hum Mol Genet.

[20] Forstner M, Lin S, Yang X (2022). High-content Screen Identifies Cyclosporin A as a Novel ABCA3-specific Molecular Corrector. Am J Respir Cell Mol Biol.

[21] Deterding R, Griese M, Deutsch G, et al. Study design of a randomised, placebo-controlled trial of nintedanib in children and adolescents with fibrosing interstitial lung disease. ERJ Open Res; 7. 10.1183/23120541.00805-2020 PMC8215331 34164554

[22] Schulz KF, Altman DG, Moher D (2011). CONSORT 2010 statement: updated guidelines for reporting parallel group randomized trials. Ann Intern Med.

